# The impact of individualized short-term prehabilitation on perioperative complications and inflammatory markers in pancreaticoduodenectomy: a single-center historical cohort study

**DOI:** 10.3389/fmed.2026.1741597

**Published:** 2026-04-16

**Authors:** Shupeng Wang, Hongbo Li, Lukang Teng, Guoliang Yao, Yonggang Fan

**Affiliations:** Department of General Surgery, The First Affiliated Hospital of Henan University of Science and Technology, Luoyang, Henan, China

**Keywords:** inflammatory markers, pancreaticoduodenectomy, perioperative complications, prehabilitation, quality of life, SF-36

## Abstract

**Background:**

Prehabilitation has emerged as a promising strategy to enhance physiological reserve and improve surgical outcomes. This study aimed to evaluate the impact of a short-term prehabilitation program on perioperative complications and inflammatory responses in patients undergoing pancreaticoduodenectomy (PD).

**Methods:**

Consecutive patients scheduled for PD between October 2024 and October 2025 were assessed for eligibility for the prehabilitation program. The intervention group underwent a minimum two-week, individualized, multimodal prehabilitation program involving physical exercise, nutritional optimization, and psychological support. A historical control cohort was established for comparison, comprising patients who underwent PD without prehabilitation between July 2023 to September 2024. Postoperative outcomes, including postoperative pancreatic fistula (POPF), delayed gastric emptying (DGE), length of hospital stay, readmission rates, mortality, and inflammatory markers (white blood cell count, C-reactive protein [CRP], and neutrophil -to-lymphocyte ratio[NLR]), were systematically recorded.

**Results:**

A total of 63 patients successfully completed the prehabilitation program. Significant improvements were observed in functional capacity, evidenced by increased 6-min walk distance (6MWD) (from 505.84 ± 65.95 m to 546.30 ± 56.49 m; *p* < 0.05) and forced expiratory volume in 1 s (FEV1%) (from 76.44 ± 4.27% to 81.23 ± 3.86%; *p* < 0.05). Quality of life (QoL) scores, assessed by the SF-36 questionnaire, also demonstrated significant enhancement. Compared to the control group, the prehabilitation group exhibited a attenuated systemic inflammatory response, with significantly lower white blood cell counts, CRP levels, and NLR on postoperative days 1 and 3 (*p* < 0.05). No statistically significant differences in the rates of clinical complications—including POPF, pleural effusion, intra-abdominal infection, reoperation, and Clavien-Dindo grade ≥III complications—were observed between the prehabilitation group and the control group, either before or after 1:1 propensity score matching (all *p* > 0.05). Supplementary analysis of the intervention cohort found no significant differences in postoperative outcomes between full-adherence (*n* = 40) and moderate-adherence (*n* = 23) subgroups (*p* > 0.05).

**Conclusion:**

Short-term prehabilitation attenuates the perioperative blood cell and CRP response and improves functional capacity and quality of life, but it does not significantly reduce postoperative complication rates in this cohort.

## 1 Introduction

Pancreaticoduodenectomy (PD) is a major surgical procedure for periampullary malignancies. However, it is associated with a high incidence of postoperative complications, such as postoperative pancreatic fistula (POPF), delayed gastric emptying (DGE), and intra-abdominal infections, which significantly compromise patient recovery (1, 2). Despite continuous technical refinements, morbidity rates following PD remain a substantial clinical challenge. Reported incidences of POPF reach 15–20%, while DGE and intra-abdominal infections also occur at considerable rates (3). These complications lead to prolonged hospitalization, increased healthcare costs, and higher mortality.

The development of postoperative complications is multifactorial, with the activation of systemic inflammatory responses recognized as a key contributor. Surgical trauma induces the release of inflammatory mediators, including elevated white blood cell counts and C-reactive protein (CRP), which can exacerbate tissue damage and predispose patients to complications (4). Early postoperative levels of inflammatory markers, such as CRP and the neutrophil-to-lymphocyte ratio (NLR), have been positively correlated with the occurrence of POPF and intra-abdominal infections (5). Therefore, mitigating the postoperative inflammatory response represents a potential strategy for reducing complication rates.

Prehabilitation, a multimodal preoperative intervention aimed at enhancing physiological reserve, has shown promise in improving surgical outcomes (6–8). Nonetheless, evidence regarding its application in patients undergoing PD is limited. While longer-term prehabilitation programs have demonstrated benefits, the efficacy of shorter interventions remains unclear (9). Thus, this study was designed to conduct a single-center historical cohort comparison to evaluate the impact of a short-term, individualized, multimodal prehabilitation program on postoperative complications, inflammatory markers, and quality of life (assessed via the SF-36 questionnaire) in patients scheduled for PD.

## 2 Materials and methods

### 2.1 Study design and patient selection

This study was a single-center historical cohort study conducted at a tertiary class hospital from October 2024 to October 2025. The study protocol was approved by the Institutional Review Board of our hospital (Number: 2025-1478). The study population comprised consecutive patients aged >18 years who were planned for PD and had a World Health Organization (WHO) Performance Status of 0 or 1. Patients were excluded for the following reasons: perioperative steroid use, planned palliative resection, incomplete medical records, reconstruction via pancreaticogastrostomy or total pancreatectomy, physical disabilities precluding exercise participation, or anticipated poor compliance. A historical control cohort consisted of patients who underwent PD from July 2023 to September 2024 and received conventional perioperative care without prehabilitation. All patients completed the quality of life questionnaire as part of standard clinical care, regardless of whether they were in the prehabilitation or standard care group.

Perioperative pathways were stable and consistent throughout the entire study period, with no changes in perioperative management, during the conduct of this study. All patient data were anonymized to protect confidentiality.

### 2.2 Prehabilitation intervention

Patients assigned to the prehabilitation group underwent a multimodal, personalized 2-week preoperative intervention, which included the following components:

#### 2.2.1 Nutritional support

Patient nutritional status was evaluated with the Nutritional Risk Screening 2002 (NRS 2002). Individuals who qualified for nutritional intervention were those meeting one or more of these criteria: (1) NRS 2002 score≥3; (2) > 5% weight loss in the past 6 months; (3) BMI < 18.5 kg/m^2^; or (4) serum albumin <30 g/L.

For at-risk patients, a clinical dietitian developed a personalized nutritional support plan that included oral nutritional supplements and dietary counseling. A dedicated dietitian conducted one-on-one follow-up and plan adjustments three times a week (on the 3rd, 7th, and 12th days of the intervention) based on the patient’s actual dietary intake and subjective feedback. In line with ESPEN guidelines, we prescribed a target protein intake of 1.5 g/kg (adjusted for obesity), and supplemented with a standardized whey protein product when patients’ dietary intake failed to meet this target; patients were also given standardized instructions to take the supplement within 1 h after exercise to optimize muscle protein synthesis. A daily caloric target of 25 ~ 35 kcal/kg was set for all patients, and this standard nutritional prescription was individually tailored for patients with diabetes mellitus to match their specific metabolic needs.

#### 2.2.2 Psychological intervention

A psychiatrist assessed patients for anxiety, depression, or psychological distress. Individuals identified with psychological concerns received supportive counseling from nurses specialized in mental health to alleviate preoperative stress.

#### 2.2.3 Exercise training

The exercise regimen was implemented in the form of daily continuous training (no intermittent rest days) throughout the prehabilitation course, with a single training session duration of 45–60 min and a total of 14 training sessions completed before surgery; the specific content included:

The exercise regimen included: Aerobic Exercise: Brisk walking for 30 min per session. Resistance Training: Elastic band exercises, comprising 3 sets of 10–15 repetitions per session. Breathing Exercises: Use of an incentive spirometer for 15–20 min per session, 3–5 times daily, to strengthen respiratory muscles.

This prehabilitation intervention was delivered in the inpatient ward under the professional supervision of dedicated nurses, combining in-ward centralized training with personalized remote guidance for individual exercise sessions. We established a private WeChat group for daily adherence monitoring and follow-up communication: participants were required to sign in for in-ward training and upload video records of individual exercise sessions to the group for verification. A dedicated research nurse supervised the group on a daily basis, reviewed all submission records, and quantified the completion of the intervention protocol. Based on this daily monitoring, protocol compliance was categorized as full (>75% of sessions completed), moderate (50–75%), or poor (<50%), with patients who had poor compliance excluded from the final analysis.

### 2.3 Data collection

Preoperative data included demographics, comorbidities (hypertension, diabetes), laboratory values (total bilirubin, direct bilirubin, albumin, CA19-9, CA125), and functional measures (6-min walk distance [6MWD], forced expiratory volume in 1 s [FEV1%]). Postoperative outcomes recorded were: complications such as POPF, hemorrhage, DGE, and bile leak, graded according to the Clavien-Dindo classification. Inflammatory markers (white blood cell count, neutrophils, lymphocytes, platelets, procalcitonin[PCT], CRP) were measured preoperatively and on postoperative days 1, 3, 5, and 7. The NLR and platelet-to-lymphocyte ratio (PLR) were also calculated.

### 2.4 Outcomes

The primary outcome of this study was surgery-related complications, including POPF, post-pancreaticoduodenectomy hemorrhage (PPH), and DGE.

Secondary outcomes included: (a) Non-surgery-related complications, such as pleural effusion, pneumonia, and atelectasis; and (b) 30-day hospital readmission rate. Other outcomes included: (a) Endurance time, measured by constant work-rate cycling exercise testing at 80% of peak oxygen uptake; (b) 6-min walk distance (6MWD); and (c) Quality of life.

Postoperative complications were defined based on established international criteria: POPF (10), PPH (11), DGE, and bile leak (12). Complication severity was classified using the Clavien-Dindo system (13).

Quality of life was evaluated using the 36-Item Short Form Health Survey (SF-36), which scores eight health domains on a scale from 0 to 100, with higher scores indicating better status (14). Functional capacity was assessed by the 6-min walk distance (6MWD). Patients were instructed to avoid strenuous activity beforehand and walked as far as possible along a 50-meter corridor under supervision. The test was performed twice, and the average distance was recorded.

### 2.5 Statistical analysis

Statistical analyses were performed using R (version 4.2.1). Normally distributed continuous variables are presented as mean ± SD and compared using Student’s *t*-test; categorical variables as *n* (%) and compared using chi-square or Fisher’s exact test. For repeated measurements of inflammatory markers (preoperative and postoperative days 1, 3, 5, 7), a two-way repeated measures ANOVA was used to evaluate the main effects of group, time, and their interaction. Missing values were handled by multiple imputation (5 iterations) with predictive mean matching, incorporating baseline characteristics and other time-point measurements of the same marker. To reduce confounding, propensity score matching (PSM) was performed: propensity scores were estimated via logistic regression including age, sex, BMI, preoperative nutritional score, and serum albumin; prehabilitation and control patients were matched 1:1 using nearest-neighbor matching without replacement (caliper width 0.02). Covariate balance was assessed by standardized mean differences (SMD < 0.1 indicating good balance). After matching, univariate regression analyses on the matched cohort were conducted. For continuous outcomes, mean differences with 95% CIs were reported; for binary outcomes, odds ratios (ORs) and risk differences with 95% CIs were reported. A two-sided *p* < 0.05 was considered statistically significant.

## 3 Results

### 3.1 Baseline characteristics

**Figure 1:**
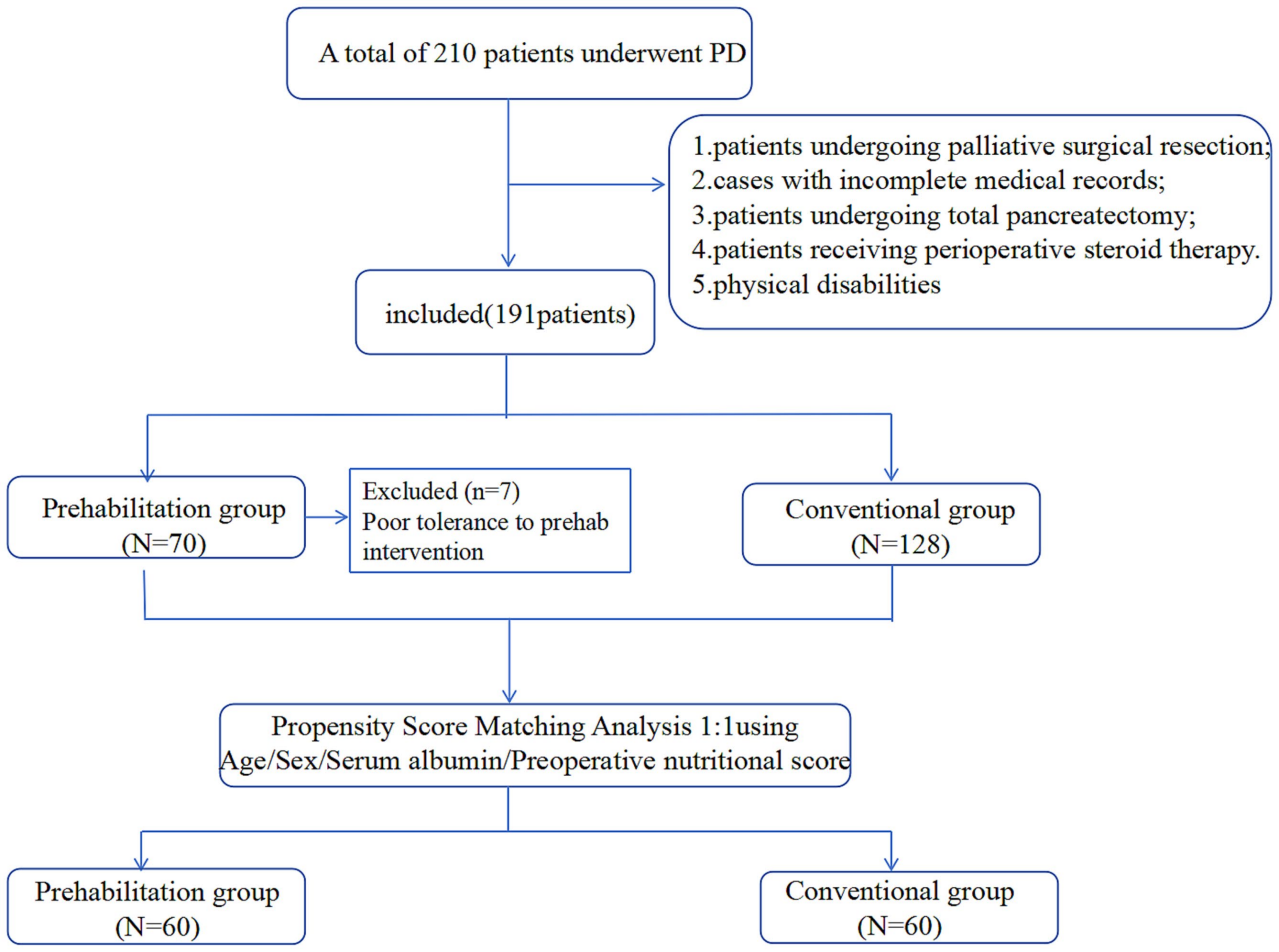
Patient flowchart.

A total of 70 patients were enrolled in the prehabilitation group. After excluding seven patients due to poor compliance, 63 patients were included in the final analysis. A historical control cohort of 128 patients who underwent PD between July 2023 and September 2024 served as the control group (Figure 1).

Baseline characteristics are detailed in Table 1. Baseline demographic and clinical characteristics, including gender, age, BMI, ASA classification, CA125, CA199, diabetes, abdominal surgery history, preoperative nutritional score, cardiovascular disease, and pathological type, were comparable between the two groups (all *p* > 0.05). Only preoperative serum albumin was significantly lower in the prehabilitation group (32.1 ± 4.1 vs. 34.1 ± 5.0 g/L, *p* = 0.006).

**Table 1:** Patient characteristics in prehabilitation and conventional group.

Variables	Prehabilitation group (*N* = 63)	Conventional group (*N* = 128)	Total group (*N* = 191)	Test statistic	*p*
Male/Female	42/21	96/32	138/53	Z = 1.22	0.222
Age (years)	60.7 ± 9.3	62.5 ± 9.0	61.9 ± 9.2	t = −1.32	0.189
BMI (kg/m^2^)	23.3 ± 3.3	22.8 ± 3.1	23.0 ± 3.2	t = 1.03	0.302
ASA classification (I-II/III-IV)	57/6	114/14	171/20	Z = 0.09	0.931
CA125 (U/mL)	16.8 (9.8–28.5)	15.9 (9.2–26.7)	16.2 (9.4–27.3)	Z = 0.41	0.682
CA199 (U/mL)	187.9 (56.3–423.5)	175.2 (51.8–408.7)	179.6 (53.2–412.9)	Z = 0.28	0.779
Diabetes (Yes/No)	8/55	15/113	23/168	Z = 0.22	0.826
Abdominal surgery history (Yes/No)	13/50	27/101	40/151	Z = 0.07	0.947
Preoperative nutritional score				χ^2^ = 1.70	0.638
Score 1	10 (15.9%)	19 (14.8%)	29 (15.2%)	Z = 0.19	
Score 2	25 (39.7%)	55 (43.0%)	80 (41.9%)	Z = −0.43	
Score 3	24 (38.1%)	42 (32.8%)	66 (34.6%)	Z = 0.72	
Score 4	4 (6.3%)	12 (9.4%)	16 (8.4%)	Z = −0.71	
Cardiovascular disease (Yes/No)	6/57	12/116	18/173	Z = 0.04	0.965
Serum albumin (g/L)	32.1 ± 4.1	34.1 ± 5.0	33.4 ± 4.8	t = −2.78	0.006
Pathological type, *n* (%)				χ^2^ = 0.80	0.669
Pancreatic adenocarcinoma	18 (28.57)	32 (25.00)	50 (26.2%)	Z = 0.53	
Cholangiocarcinoma	29 (46.03)	58 (45.31)	87 (45.5%)	Z = 0.09	
Ampullary carcinoma	16 (25.39)	38 (29.68)	54 (28.3%)	Z = −0.62	

ASA classification, American society of anesthesiologists classification; BMI, body mass index.

**Figure 2:**
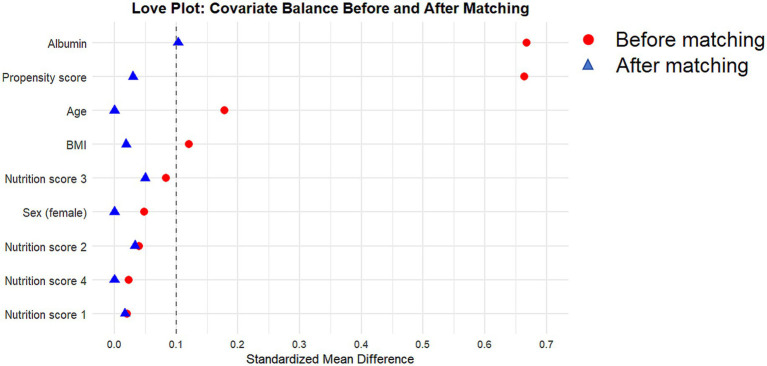
Love plot of covariate balance before and after propensity score matching.

To reduce the impact of potential confounding bias, Table 2 shows the characteristics of the two groups after 1:1 propensity score matching, resulting in 60 patients in each group. Following matching, all baseline variables were well balanced between the prehabilitation and conventional group. The propensity score distance was comparable between groups after matching (0.38 ± 0.15 vs. 0.37 ± 0.14, standardized difference = 0.030), and the love plot is presented in Figure 2.

**Table 2:** Standardized mean difference values before and after propensity score matching.

Variables	Before matching		Standardized mean difference	After matching		Standardized mean difference
	Prehabilitation group (*N* = 63)	Conventional group (*N* = 128)		Prehabilitation group (*N* = 60)	Conventional group (*N* = 60)	
Male/Female	43/20	95/33	0.048	41/19	41/19	0.000
Age (years)	60.7 ± 9.3	62.5 ± 9.0	0.178	61.5 ± 9.4	61.6 ± 9.0	0.000
BMI (kg/m^2^)	23.3 ± 3.3	22.8 ± 3.1	0.121	23.1 ± 3.2	23.1 ± 3.0	0.019
Serum albumin (g/L)	32.1 ± 4.1	34.1 ± 5.0	0.668	32.6 ± 4.2	33.5 ± 4.5	0.103
Preoperative nutritional score 1	10 (15.9)	19 (14.8)	0.020	10 (16.7)	9 (15.0)	0.017
Preoperative nutritional score 2	25 (39.7)	55 (43.0)	0.040	24 (40.0)	26 (43.3)	0.033
Preoperative nutritional score 3	24 (38.1)	42 (32.8)	0.084	22 (36.7)	20 (33.3)	0.050
Preoperative nutritional score 4	4 (6.3)	12 (9.4)	0.023	4 (6.7)	5 (8.3)	0.000
Propensity score (distance)	0.48 ± 0.22	0.29 ± 0.18	0.664	0.38 ± 0.15	0.37 ± 0.14	0.030

### 3.2 Impact of prehabilitation on functional and psychological outcomes

**Figure 3:**
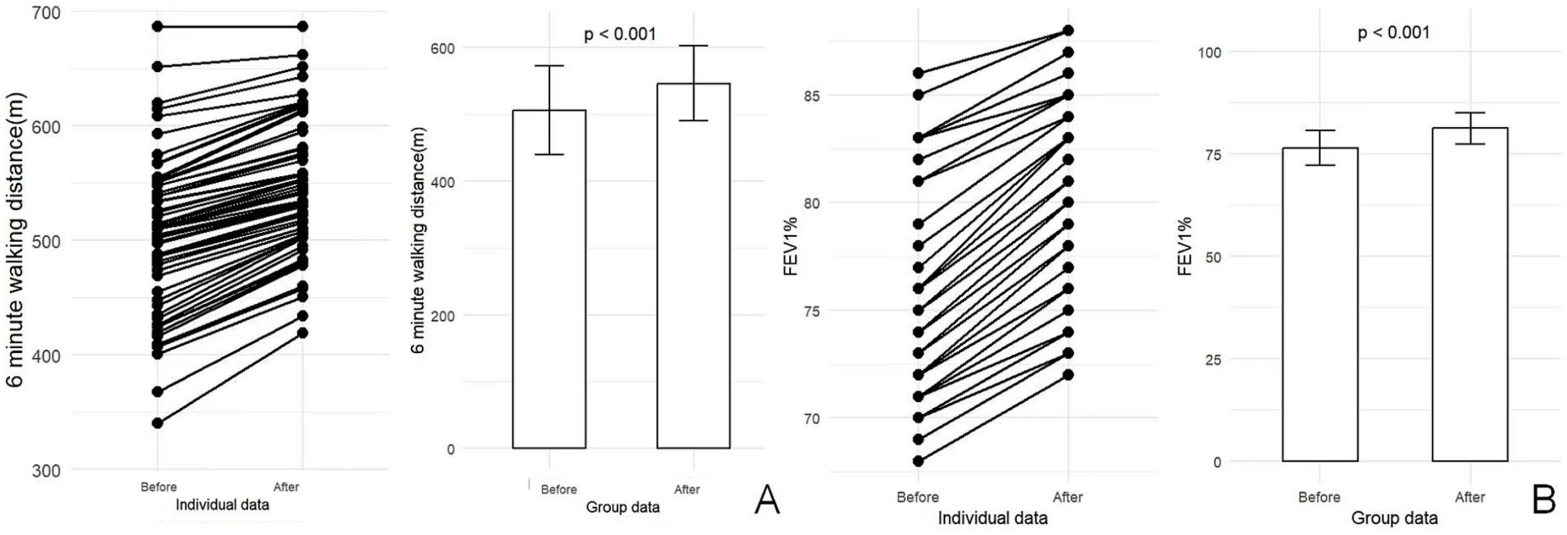
The changes in 6-minute walk distance (6MWD) distance and forced expiratory volume in percentage (FEV%) before and after prehabilitation among patients who underwent the intervention. **(A)** The variations in 6MWD distance for patients before and after prehabilitation. **(B)** The corresponding changes in FEV% before and after the prehabilitation program.

Table 3 demonstrates the significant beneficial effects of prehabilitation on physical and psychological status in the intervention group. After prehabilitation, significant reductions were observed in HAD anxiety and total HAD scores (*p* < 0.001 and *p* = 0.036, respectively), whereas HAD depression scores showed no significant change (*p* = 0.279). Physical function was also markedly improved. The intervention group exhibited a significant increase in 6MWD (546.30 ± 56.49 m vs. 505.84 ± 65.95 m; Figure 3A). Respiratory function improved significantly, with higher forced vital capacity (FVC; 2.88 ± 0.69 L vs. 2.49 ± 0.56 L) and FEV1% (81.23 ± 3.86% vs. 76.44 ± 4.27%; Figure 3B). Additionally, pulmonary function parameters (FVC and FEV1%) were significantly improved post-intervention. Furthermore, the radar chart shows that prehabilitation comprehensively improved health-related quality of life, with statistically significant enhancements observed in physical function, overall physical function, mental health, vitality (*p* < 0.05).

**Table 3:** The impact of prehabilitation interventions on physical function.

Variables	Prehabilitation group		Paired *t* value	*P* value
	At baseline (pre-prehabilitation)	Post-prehabilitation		
HAD anxiety	7.87 ± 3.18	5.74 ± 1.90	4.552	<0.001
HAD depression	6.90 ± 2.58	6.57 ± 2.80	0.694	0.279
HAD total score	14.77 ± 4.08	12.31 ± 3.12	3.801	0.036
6MWT (s)	505.84 ± 65.95	546.30 ± 56.49	3.698	<0.001
Endurance time (s)	336.96 ± 27.05	656.54 ± 49.69	23.956	<0.001
Total physical SF-36 subscale	51.26 ± 2.94	75.11 ± 2.18	3.801	<0.001
FVC (L)	2.49 ± 0.56	2.88 ± 0.69	3.508	<0.001
FEV 1%	76.44 ± 4.27	81.23 ± 3.86	6.606	<0.001

### 3.3 Comparison of perioperative complications

Propensity score analysis showed no statistically significant differences between the prehabilitation group and the control group in the rates of all recorded postoperative complications—including POPF, pleural effusion, intra-abdominal infection, mortality, and pneumonia—either before or after 1:1 matching (all *p* > 0.05; Table 4). No significant differences were found in postoperative gastrointestinal recovery (time to oral intake, gastrointestinal recovery, nasogastric tube removal, ambulation) between groups (Table 5).

**Table 4:** Postoperative outcomes of those patients in prehabilitation and conventional group.

Variables	Without propensity score matching		*P*	With propensity score matching		*P*	Effect size (95% CI)	Odds ratio (95% CI)
	Prehabilitation group (*N* = 63)	Conventional group (*N* = 128)		Prehabilitation group (*N* = 60)	Conventional group (*N* = 60)			
Intraoperative transfusion (Yes/No)	6/57	11/117	0.832	4/56	6/54	0.509	−3.3% (−13.2, 6.5%)	0.62 (0.17, 2.41)
Operation time (min)	225.05 ± 62.13	219.21 ± 59.55	0.613	216.12 ± 57.88	223.32 ± 62.10	0.512	−7.20 (−28.9, 14.5)	
Estimates blood loss (ml)	212.73 ± 133.78	248.75 ± 158.69	0.122	212.85 ± 37.37	243.02 ± 101.22	0.032	−30.2 (−57.9, −2.4)	
Hospital stay (days)	14.71 ± 3.37	15.34 ± 4.25	0.306	14.01 ± 2.47	15.17 ± 4.07	0.064	−1.16 (−2.38, 0.06)	
Postoperative hospital stay (days)	10.94 ± 3.19	11.60 ± 4.31	0.255	10.23 ± 2.25	11.43 ± 3.82	0.038	−1.20 (−2.34, −0.06)	
Type of surgery								
Open	10 (15.87)	24 (18.75)	0.887	9 (15.00)	8 (13.33)	0.817	1.7% (−10.8, 14.2%)	1.15 (0.41, 3.20)
Laparoscopic	47 (74.60)	92 (71.88)		46 (76.67)	45 (75.00)		1.7% (−13.7, 17.0%)	1.10 (0.48, 2.52)
Conversion to open	6 (9.52)	12 (9.37)		5 (8.33)	7 (11.67)		−3.3% (−14.1, 7.4%)	0.69 (0.21, 2.30)
Hospital readmission <30 days	5 (7.93)	11 (8.59)	0.877	4 (6.67)	6 (10.00)	0.101	−3.3% (−13.2, 6.5%)	0.62 (0.17, 2.41)
Comprehensive complication index	33.76 ± 3.03	34.04 ± 2.62	0.003	34.20 ± 3.01	35.05 ± 2.47	0.094	−0.85 (−1.85, 0.15)	
C-D classification ≥3	18/45	43/85	0.484	16/44	18/42	0.685	−3.3% (−19.4, 12.8%)	0.85 (0.38, 1.88)
Non-surgical complication								
Pleural effusion	6 (9.5)	14 (10.9)	0.764	5 (8.33)	7 (11.67)	0.685	−3.3% (−14.1, 7.4%)	0.69 (0.21, 2.30)
Pneumonia/atelectasis	4 (6.3)	9 (7.0)	0.860	4 (6.67)	5 (8.33)	0.729	−1.7% (−11.1, 7.8%)	0.79 (0.20, 3.08)
Thromboembolic	1 (1.6)	3 (2.3)	0.731	1 (1.67)	1 (1.67)	1.000	0.0% (−4.6, 4.6%)	1.00 (0.06, 16.4)
Surgical complication								
POPF (Yes/No)	6/57	16/112	0.545	5/55	8/52	0.378	−5.0% (−16.1, 6.1%)	0.59 (0.18, 1.92)
PPH (Yes/No)	9/54	20/108	0.808	9/51	8/52	0.793	1.7% (−10.8, 14.2%)	1.15 (0.41, 3.21)
DGE (Yes/No)	4/59	9/119	0.860	4/56	3/57	0.697	1.7% (−6.7, 10.1%)	1.36 (0.29, 6.34)
Intra-abdominal infection (Yes/No)	8/55	20/108	0.591	7/53	9/51	0.591	−3.3% (−15.5, 8.8%)	0.75 (0.26, 2.16)
Additional reoperation, *n* (%)	4 (6.35)	9 (7.03)	0.806	4 (6.67)	3 (5.00)	0.697	1.7% (−6.7, 10.1%)	1.36 (0.29, 6.36)

C-D classification, Clavien-Dindo classification; POPF, Postoperative Pancreatic Fistula; PPH, post pancreaticoduodenectomy hemorrhage; DGE, Delayed Gastric Emptying.

**Table 5:** Recovery milestones after PD: prehabilitation versus conventional care, with and without propensity score matching.

Variables	Without propensity score matching		*P*	With propensity score matching		*P*	Mean difference (95% CI)
	Prehabilitation group (*N* = 63)	Conventional group (*N* = 128)		Prehabilitation group (*N* = 60)	Conventional group (*N* = 60)		
The time to first flatus (h)	34.47 ± 15.14	37.85 ± 15.47	0.154	34.16 ± 14.39	36.70 ± 15.97	0.354	−2.54 (−8.04, 2.96)
The time to removal of nasogastric tube (h)	57.93 ± 23.65	57.59 ± 24.19	0.926	53.58 ± 20.06	56.28 ± 24.75	0.513	−2.70 (−10.85, 5.45)
The time to initiation of regular diet (h)	42.15 ± 10.42	45.66 ± 11.28	0.040	42.83 ± 10.85	44.86 ± 11.05	0.287	−2.03 (−5.99, 1.93)
The time to first ambulation (h)	34.65 ± 7.84	35.79 ± 8.84	0.385	34.58 ± 7.70	35.93 ± 7.97	0.348	−1.35 (−4.18, 1.48)

**Figure 4:**
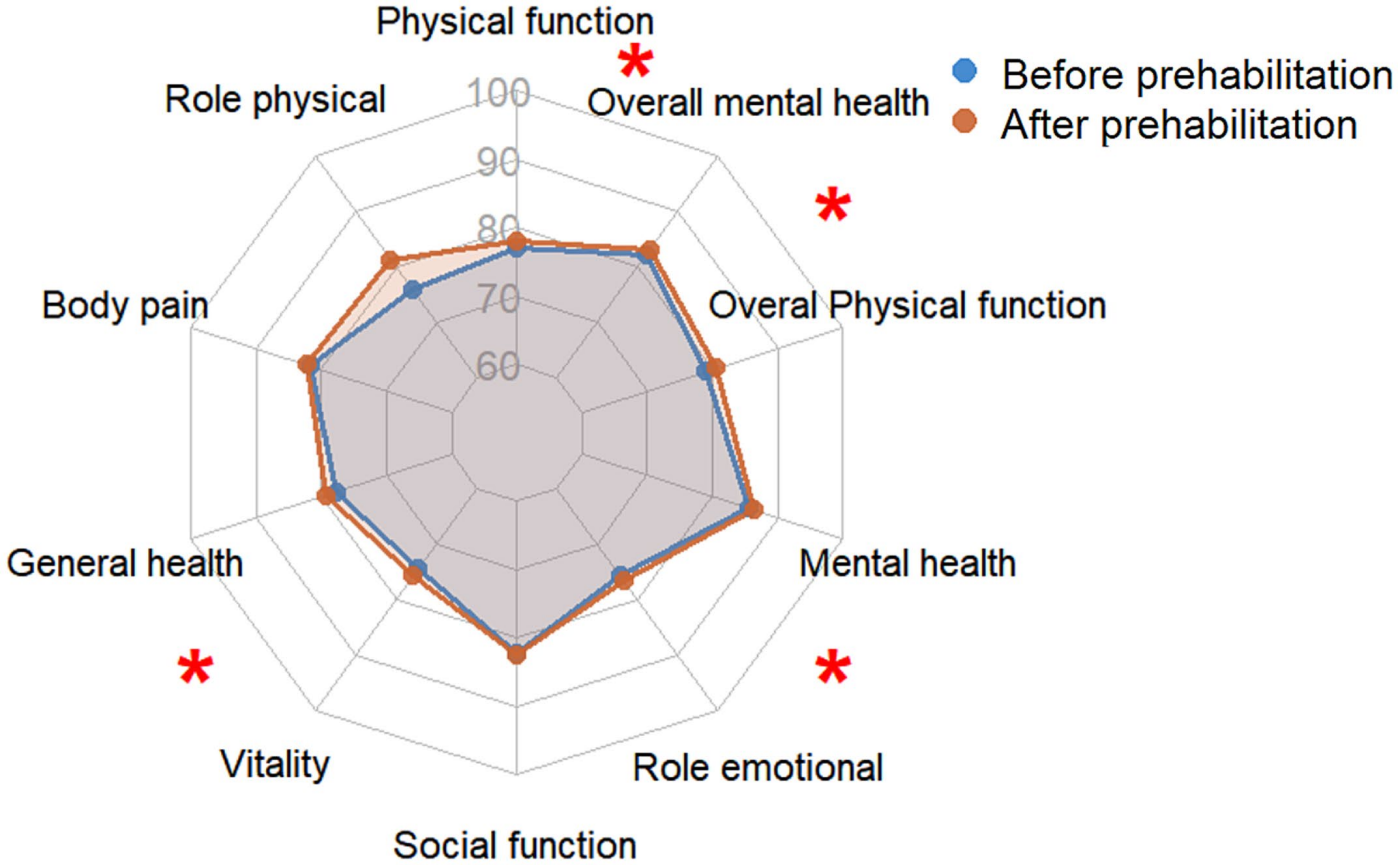
Radar graphs demonstrating changes in quality-of-life scores in prehabilitation group of patients undergoing PD.

Poor compliance with prehabilitation was mainly attributed to a lack of effective supervision, insufficient patient awareness and perceived functional gains, and physical limitations such as pain and jaundice, which made it objectively difficult for them to complete the exercise and nutritional intervention as required. A total of 7 patients were excluded from the final analysis due to poor compliance (<50% of sessions completed), which may introduce potential selection bias in the study (Figure 4).

To further explore the impact of adherence on study outcomes, we conducted a stratified subgroup analysis on the 63 included patients according to pre-established compliance criteria. No significant differences were observed in postoperative outcomes and inflammatory marker levels between two subgroups.

### 3.4 Comparison of postoperative inflammatory markers

**Figure 5:**
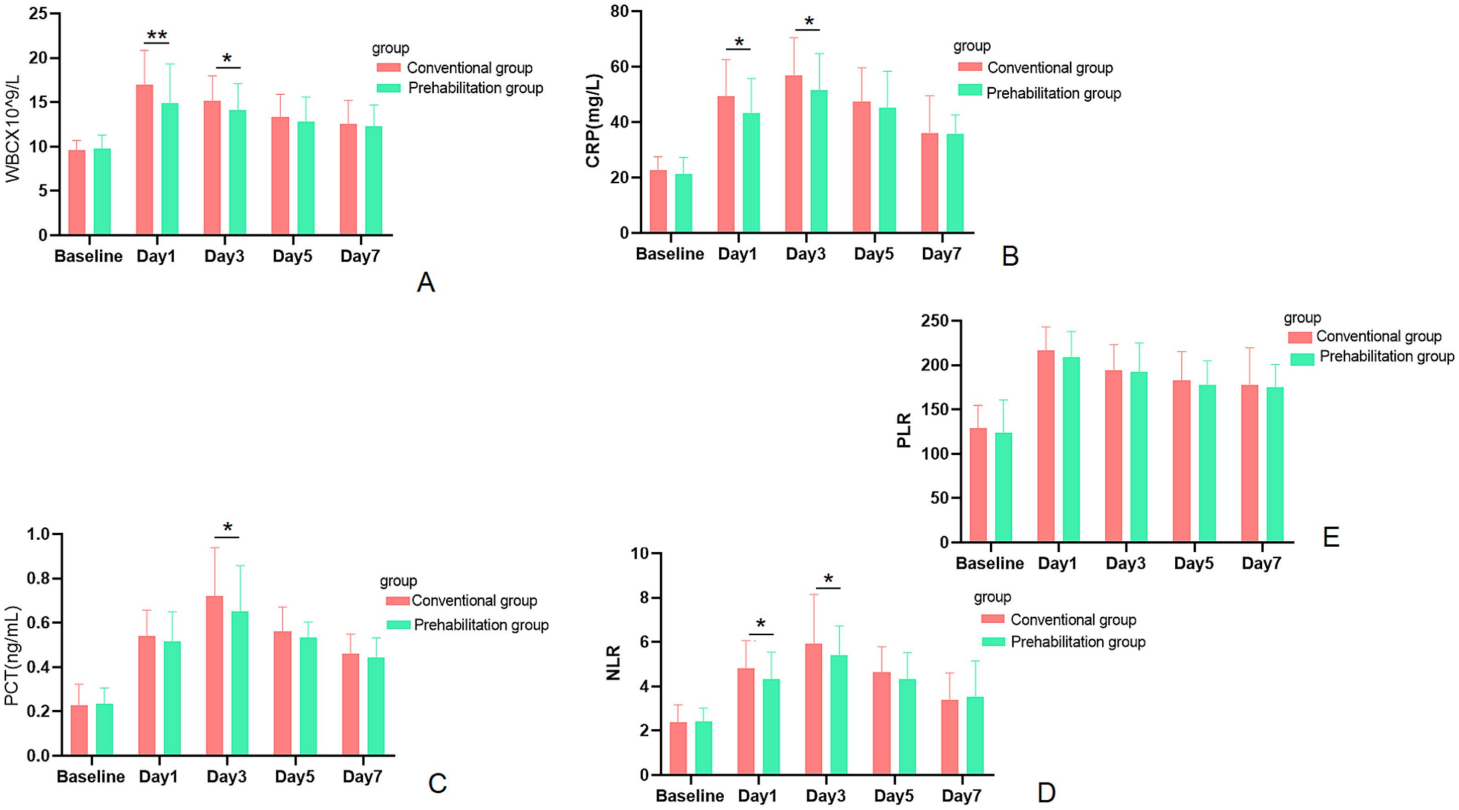
The changes in perioperative inflammatory markers in the prehabilitation group and the conventional group. **(A)** The changes in WBC at different time points. **(B)** The changes in CRP at different time points. **(C)** The changes in PCT at different time points. **(D)** The changes in NLR at different time points. **(E)** The changes in PLR at different time points.

As shown in Figure 5, inflammatory markers differed significantly between the groups at specific postoperative time points. Compared to the control group, the prehabilitation group had significantly lower WBC counts on postoperative day 1 (POD1; *p* = 0.002) and POD3 (*p* = 0.0312). CRP levels were also significantly lower in the prehabilitation group on POD1 (*p* = 0.013) and POD3 (*p* = 0.022). The NLR ratio was significantly reduced on POD3 (*p* = 0.017). In contrast, no significant intergroup differences were observed in PCT or PLR levels at any time point.

## 4 Discussion

This study found that short-term preoperative prehabilitation did not significantly reduce the incidence of postoperative complications, although it could attenuate perioperative blood cell and CRP response and improves functional capacity and quality of life.

Although prehabilitation has shown potential in improving surgical outcomes, the optimal intervention protocol remains controversial (15). Excessively short programs may fail to meaningfully enhance functional capacity, while prolonged interventions risk delaying treatment and potentially promoting tumor progression (16). This notion is supported by previous studies: Carli et al. reported no significant difference in the 30-day CCI between prehabilitation and standard care groups in patients undergoing colorectal surgery (8); similarly, Gillis et al. observed that while nutritional and exercise prehabilitation shortened hospital stays, it did not significantly reduce perioperative complications (17). In contrast, Liang et al. demonstrated a notable reduction in complication rates (69.5% vs. 47.5%, *p* = 0.015) among elderly patients undergoing hernia repair after combined nutritional and exercise prehabilitation (18). Additionally, other studies have reported that prehabilitation programs lasting 4–8 weeks significantly improved physical function, muscle strength, and quality of life, highlighting the importance of intervention duration (19, 20).

The exercise component of multimodal prehabilitation is specifically designed to enhance the physiological reserve and functional capacity of the cardiopulmonary and musculoskeletal systems (21). Multiple prospective studies have confirmed that implementing exercise prehabilitation during the preoperative waiting period effectively improves patients’ functional capacity (22, 23). Furthermore, in small non-randomized trials, weekly structured exercise training programs significantly increased exercise capacity in patients with advanced colorectal cancer (24, 25). This beneficial effect is further validated by multiple meta-analyses, which have confirmed that preoperative exercise-based prehabilitation interventions not only significantly improve physical function (26, 27) but also exert a protective effect against postoperative morbidity, especially pulmonary complications (28).

Mechanistically, the efficacy of prehabilitation can be contextualized within the framework of the surgical stress response. Gillis C et al. proposed that preoperative patient status can influence surgical outcomes by mediating the surgical stress response (21). As an intervention strategy, Enhanced Recovery After Surgery (ERAS) improves surgical outcomes by attenuating this stress response, and prehabilitation serves as an important complement to ERAS by targeting preoperative physiological reserve, thereby further modulating the stress response and ultimately enhancing therapeutic efficacy (21). Dunne DF further emphasized that preoperative prehabilitation, as a core component of ERAS, can reduce preoperative anxiety, improve postoperative gastrointestinal motility, promote wound healing, and attenuate the postoperative stress response, collectively contributing to improved surgical outcomes (29).

In the present study, although the prehabilitation cohort showed no statistically significant reduction in postoperative complications. This non-significant finding may be attributed to multiple factors: first, the 2-week short-term prehabilitation may be insufficient to induce robust physiological changes that translate to a measurable reduction in PD-related complications, which are inherently high due to the complexity of the surgical procedure; second, the sample size of the study is relatively small, leading to limited statistical power to detect small but clinically meaningful differences in complication rates; third, PD is associated with a variety of severe complications, and single-center setting may limit the capture of the full spectrum of perioperative outcomes.

Notably, the prehabilitation cohort exhibited lower levels of WBC, CRP, and NLR on POD1 and POD3. This finding indicates that prehabilitation effectively modulates perioperative inflammatory response, blunting the early postoperative inflammatory peak induced by surgical trauma. The reduced inflammatory response in the prehabilitation cohort may be attributed to several interconnected mechanisms. First, prehabilitation improves physical and nutritional status, which in turn enhances immune and antioxidant function and promotes a balance between pro- and anti-inflammatory cytokines (30). Second, exercise alleviates psychological stress and reduces the secretion of stress hormones, thereby mitigating inflammatory cascades and tissue damage (31). Third, aerobic exercise enhances cardiorespiratory fitness and tissue oxygenation, reducing hypoxia-related inflammation (32). Furthermore, prehabilitation was associated with faster gastrointestinal function recovery (33), and the shorter hospital stay in the prehabilitation group may also reflect improved physiological reserve and surgical tolerance conferred by preoperative interventions (34).

This study has several limitations. First, selection bias may have been introduced by excluding seven patients with poor prehabilitation compliance; this exclusion may have enriched the cohort with more motivated and physically capable individuals, potentially overestimating the benefits of prehabilitation and masking its true effect on complication rates. To address this limitation, we conducted a supplementary exploratory analysis of the included patients by stratifying them into full-adherence and moderate-adherence subgroups, and found no significant differences in postoperative outcomes between the two subgroups. And the lack of a sensitivity analysis including these patients is a further weakness. Second, the preoperative intervention lasted only 2 weeks, which may have been insufficient to optimize functional recovery and reduce complications. Third, the sample size of the matched cohort after 1:1 propensity score matching was relatively small, which limits the statistical power of the study to detect potential small-effect differences in postoperative complication rates; minor numerical variations in complication rates between the two groups were observed, but such variations may merely stem from random differences in individual patients. Additionally, this is a historical cohort study, which inherently carries potential confounding biases that cannot be fully eliminated by PSM—such as minor discrepancies in anesthesia processes, blood transfusion strategies, and postoperative nursing protocols between the intervention period and the historical control period—and these factors may have interfered with the study results, necessitating further verification via prospective studies. Fifth, although multiple inflammatory markers were evaluated, other key mediators such as interleukin-6 (IL-6) and tumor necrosis factor-*α* (TNF-α) were not included, which limits the in-depth exploration of the inflammatory modulation mechanism of prehabilitation.

## 5 Conclusion

In summary, short-term preoperative prehabilitation did not significantly reduce the incidence of perioperative complications in patients undergoing PD. However, it could effectively attenuates the perioperative blood cell and CRP response and improves functional capacity and quality of life.

## Data Availability

The raw data supporting the conclusions of this article will be made available by the authors, without undue reservation.
